# Temporal samples of visual information guides skilled interception

**DOI:** 10.3389/fpsyg.2024.1328991

**Published:** 2024-02-26

**Authors:** Sean Müller, Bradley Beseler, Khaya Morris-Binelli, Christopher Mesagno

**Affiliations:** ^1^Centre for Smart Analytics, Federation University Australia, Ballarat, VIC, Australia; ^2^Institute of Education, Arts & Community, Federation University Australia, Ballarat, VIC, Australia; ^3^School of Health Sciences, The University of Notre Dame Australia, Fremantle, WA, Australia; ^4^Institute of Health and Sport, Victoria University, Melbourne, VIC, Australia; ^5^Institute of Health and Wellbeing, Federation University Australia, Ballarat, VIC, Australia

**Keywords:** visual-perception-action cycle, stroboscopic vision, interceptive skill, attentional focus, goal kicking

## Abstract

This study investigated whether performance of an interceptive skill requires an intact visual-perception-action cycle. Eleven skilled male Australian rules football athletes (M_age_ = 17.54, SD = 0.15) were recruited from an elite developmental pathway squad for a within-subject study. Participants were required to kick a ball directly at a goal from a 20-meter distance while wearing a pair of stroboscopic glasses. The glasses were used to create four vision conditions. Condition one kept intact the visual-perception-action cycle with uninterrupted vision of the motor skill. Three other conditions included stroboscopic vision that presented temporal samples of vision, which interrupted the perception-action cycle through progressive increases to intermittent vision occlusion of the motor skill. Goal kick error of ball position relative to a central target line within the goal and number of successful goals kicked were measured. Written report of internal and external focus of attention was also measured after each vision condition. Generalized estimating equation analysis did not reveal a significant decrement in kick target error, nor accuracy of goals scored, across normal to stroboscopic vision conditions. Performance was maintained despite a shift in attention focus from external to internal across normal to stroboscopic vision conditions. These findings have theoretical and practical implications for the visual regulation of skilled interceptive actions.

## 1 Introduction

The use of visual-perceptual information to guide action is crucial to a variety of motor skills including interception of a ball with a foot or by hand using an implement in sports (Hodges et al., 2021). Expertise and motor learning researchers are interested in understanding the mechanism that underpins visual-perception-action of these complex motor skills because they are executed in challenging contexts (Morris-Binelli and Müller, 2017). For example, a soccer penalty taker, attempting to score against the opposition goalkeeper, needs to use visual information to guide their run toward the ball and intercept it with spatiotemporal accuracy, as well as force, to launch it past the keeper into the goal to score (Tomeo et al., 2013). Knowing how the brain uses visual information to control such complex skills contributes to theoretical advancement, which will provide a strong foundation for future training and development of perceptual-cognitive-motor skills.

Visual control of action can be explained from three theoretical perception-action loops. First, open-loop control states that temporal samples of visual information in the environment guide ballistic type actions when there is no time for adjustments (Adams, 1971; Schmidt et al., 2019). Second, closed-loop control also states that temporal samples of visual information from the environment are used, but adjustments to ongoing action occur if the time constraints permit (Elliott et al., 2001, 2020). Third, continuous perception-action cycle based control states that visual-perception informs action, and on-going action further informs perception to achieve the skill goal (Magill and Anderson, 2021). It is unclear whether a seamless intact perception-action cycle that allows visual information to synchronize with on-going movement is crucial for successful completion of the skill goal. Expertise and motor learning researchers have manipulated visual information during complex *in-situ* sports skills to understand how spatiotemporal visual information is used for action.

The duration of visual information for action has been manipulated through the use vision occlusion glasses worn by the performer (Milgram, 1987). Elliott and colleagues (see Elliott and Bennett, 2020) have conducted research where intermittent visual information of a projected ball was presented during one handed catching. The main finding from these series of studies is that intermittent visual samples, rather than continuous vision, of ball flight information is sufficient to maintain catching performance. For complex whole body sports skills expertise, Abernethy et al. (2001) were the first to investigate whether temporal pick-up of visual information available prior to object flight (known as advance cues) could be used for action control. In simulated squash matches, vision of highly skilled and lesser skilled performers was occluded prior to the opponent’s racquet-ball contact. Results revealed that highly skilled players were superior to lesser skilled players at moving into court position when preparing to intercept the ball. Accordingly, Abernethy et al. (2001) provided evidence that advance visual information could be used in open-loop fashion to control complex whole-body positioning in sport. Subsequent studies that used occlusion glasses, or props to occlude pre-object flight cues, have confirmed the contribution of advance visual information, aligning with open-loop control, for positioning the body for interception in tennis return of serve (Farrow and Abernethy, 2003), cricket batting (Müller et al., 2009), and ice hockey goal tending (Panchuk and Vickers, 2009). Further, through comparison of temporal visual occlusion during early sections of object flight to a no occlusion control condition that presented all object flight, highly skilled cricket batsmen and ice hockey goaltenders could make fine adjustments to their bat or stick to intercept a ball or puck, respectively (Müller and Abernethy, 2006; Müller et al., 2009; Panchuk and Vickers, 2009). This indicates that experts can make fine adjustments based on later occurring components of complex whole-body skills, which aligns with closed-loop and perception-action cycling accounts of action control.

More recently, stroboscopic glasses that present a fixed duration of vision for 100 milliseconds, within cyclical periods of visual occlusion that vary in duration, have been used to investigate visual control of action. Fransen et al. (2017) investigated junior soccer players dribbling efficiency across a course under normal and stroboscopic vision conditions. Fransen et al. reported that faster dribblers had a greater decrement in time to course completion and ball control accuracy compared to slower dribblers when vision was increasingly restricted. Furthermore, Beavan et al. (2020) investigated whether amateur soccer players could complete a ball reception and pass test across normal and stroboscopic vision conditions. Stroboscopic vision caused a decrease in test completion time and accuracy. Both these studies indicate that for junior and amateur players, continuous visual information for action is necessary to maintain performance of dribbling or repetitive kicking sports skills. These findings could be because at such skill levels predictive capability of action is not sufficiently developed to guide and/or adjust performance. Accordingly, it is unclear whether cyclical interruptions to visual-perception-action will impede performance in skilled athletes who as mentioned earlier have superior predictive capability. In addition, it is unclear whether temporal samples of visual information can be used to guide action in closed sports skills. Therefore, further investigation of how visual-perception-action cycling influences complex skills in higher level sports players is needed.

Given that vision and proprioceptive information are used during motor skill performance, with the former the dominant sensory modality (Lee and Aronson, 1974; Elliott and Bennett, 2020), occlusion paradigms may alter in part (if not in whole) a performer’s attentional focus. External focus refers to attention that can be directed by a performer, such as a batter, to advance cues from an opponent pitcher or bowler, and by a penalty taker to the intended goal location of a soccer ball (Wulf et al., 2001). Alternatively, internal focus refers to attention that can be directed by a performer to aspects of the movement pattern used to strike or kick a ball (Wulf et al., 2001). The constrained action hypothesis predicts that internal focus of attention will de-automatize skilled movement patterns and result in a decrement in skilled performance (Wulf et al., 2001). Accordingly, studies that have manipulated performer external and internal focus by experimenter instructions of what to focus attention on report that internal focus results in decreased performance in sports skills such as golf putting (Beilock et al., 2002; Klostermann et al., 2014; An and Wulf, 2024), volleyball passing (Singh and Wulf, 2020), slalom skiing (Wulf et al., 2001), and cricket ball striking (Bull et al., 2022). More recently, in line with meshed control theory, which predicts that conscious and automatic processes can work synchronously during motor skill performance (Christensen et al., 2016), skilled golfers can switch attention from external to internal focus prior to ball-putter contact without a performance decrement (Wang et al., 2021). Accordingly, some internal focus could draw attention toward task-related proprioceptive information, which as mentioned earlier, contributes in part to motor skill performance (Herrebrøden, 2022). Stroboscopic glasses present a method where through decreased occlusion cycling and therefore increased vision, attention may partly be drawn externally, or through increased occlusion cycling and decreased vision, attention could be drawn more internally. Such a methodology presents a more direct way for the experimenter to induce internal or external attention focus compared to direct instruction of where to focus attention, which may have inherent experimental bias and inconsistency of data collection. Previous studies that manipulated internal and external attentional focus across vision and no-vision conditions have reported that complete availability of vision did not along with attention focus influence performance (Abdollahipour et al., 2016; Saemi et al., 2017). This suggests that visual information and attentional focus instructions operate independently to guide performance. However, such strictly manipulated attentional focus and vision conditions do not take into account the dynamic nature of attentional shifts that may contribute to visual control of action in expert performance (Wang et al., 2021; Herrebrøden, 2022). Having a method that can consistently create a partial focus of attention could determine how such manipulations influence skilled performance in sport skills (Wang et al., 2021; Herrebrøden, 2022).

The present study used the *set shot* kick in Australian football as the exemplar perceptual-motor skill to explore how visual information regulates action and how attentional focus might influence performance. The set shot, a similar skill to an National Football League punt where the ball is guided by one hand to be intercepted with their foot and kicked toward goal, is a key goal scoring option where an offensive player catches the ball (known as a mark), and from a standing start, runs toward, and kicks at the goal over an opposition player standing where the ball was marked (Mesagno and Mullane-Grant, 2010; Blair et al., 2020). The objective is to kick the ball between the two center posts to score six points, with a single point resulting when the ball is kicked between either of the two outer scoring sections. A complete miss of all sections results in a score of zero. We created conditions where the performer (kicker) wore a pair of stroboscopic glasses, then executed set shots under normal uninterrupted vision, and increasing cyclical occlusion-to-vision conditions. Based upon the literature discussed above, three hypotheses were formulated. First, if temporal samples of visual information were sufficient to control action, kicking accuracy would not decrease across uninterrupted (normal) to stroboscopic vision conditions. Second, if continuous perception-action cycling was critical for visual control of action, kicking accuracy would decrease from uninterrupted (normal) to stroboscopic vision conditions. Third, a shift in written report of attention from external to internal focus would occur as vision occlusion increased and this would result in a decrease in kicking accuracy.

## 2 Materials and methods

### 2.1 Participants

Eleven skilled male footballers were recruited from an Australian rules football elite developmental pathway team. These participants formed a current group of emerging expert players in the region of their state, which is consistent with the sport expertise literature (Connor et al., 2018). The mean age of players was 17.54 years (SD = 0.15, age range: 17–18 years). Based upon our generalized estimating equation analysis (see data analysis section), an *a-priori* power analysis conducted in G*Power (Version 3.1.9.7) with *α* = 0.05, 80% power, and 95% confidence interval, for a repeated measures analysis, indicated that 40 trials per participant (440 trials in total) could detect a small effect size. Participants volunteered for the study and provided written informed consent, with ethics approval obtained from the lead institution (permit number E21-004).

### 2.2 Materials and procedure

Participants wore Senaptec stroboscopic glasses (Model 26125) to create four vision conditions and completed set shot kicks at goal (see Mitroff et al., 2013 for details on glasses). Condition 1 (hereafter called normal vision) consisted of the glasses being worn, but were not activated, which provided clear vision of the run-up, kick action, and goal. Conditions 2, 3, and 4 consisted of the glasses being activated to Strobe settings 1 (67 ms), 5 (344 ms), and 8 (900 ms), occlusion time, respectively. The strobe feature involved the glasses cycling intermittently from a clear state that provided vision of the environment to an opaque state that occluded vision, with the clear state consistently 100 ms, and the opaque state set at 67 ms, 344 ms, and 900 ms for settings 1, 5, and 8, respectively (Mitroff et al., 2013). These pre-determined settings were selected by the experimenters to progressively increase cyclical intermittent vision occlusion time.

**Figure 1:**
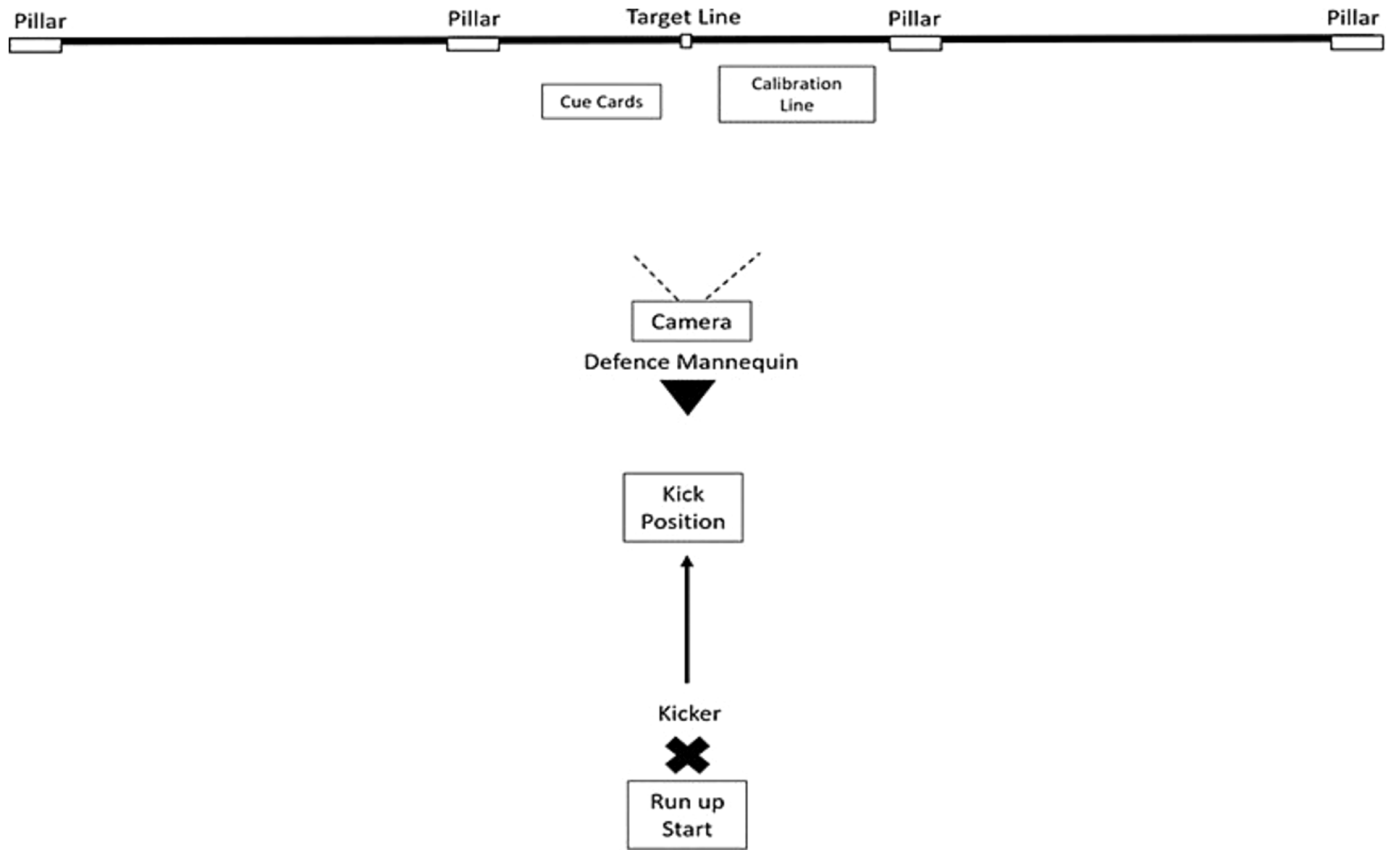
Schematic diagram (aerial view) of the experimental set-up for the study. The participant started in a stationary position (position ×), ran toward, and kicked the ball over a mannequin toward the vertical target line. The inside pillars represented the goal posts, and the outside pillars represented the point posts. The camera captured the ball position relative to the target line and whether a goal or point was scored. The calibration line was used to scale error measurements and cue cards were used to record condition/trial numbers.

During the task, participants performed a set shot kick with an Australian football (oval shaped ball) at a vertical target ribbon located in the middle of a simulated Australian football goal (see Figure 1 for experimental set-up). Participants were instructed to kick at the ribbon using their normal set shot routine. The rationale for these instructions is that recent literature indicates that attentional focus is dynamic during the preparation to execution time course of motor skills, rather than being fixed as external or internal focus *per se* (Wang et al., 2021; Herrebrøden, 2022). Participants were permitted a 10-meter run-up and then kicked over an adult size mannequin that was positioned 20 meters from and directly in front of the goal in an indoor sports center. The mannequin simulated an opposition player standing in the position where the mark was taken. In a game, set shot kicks can be executed from directly in front of the goal face, so our task was representative of a typical Australian football game (Mesagno and Mullane-Grant, 2010; Blair et al., 2020). A Go-Pro camera (model HERO9 Black) sampling at 50 frames per second was positioned behind the mannequin to capture where the kicked ball entered the scoring area relative to the vertical target. Condition and trial number cue cards, as well as a calibration distance measure were filmed in the camera field of view. Footage was imported into Kinovea (version 0.8.15) software where a calibrated frame-by-frame analysis was conducted to calculate target error and to determine whether a goal or point was scored.

Prior to beginning the task, participants completed an Australian football specific physical warm-up and completed five set shot kicks without the stroboscopic glasses. Thereafter, participants completed 10 kicking trials in a block under each of the four vision conditions, which were counterbalanced across participants. The experimenter manually changed the settings on the glasses to correspond to the relevant vision condition prior to each new vision condition. After each kick, participants walked back to their starting position and were handed the ball by the experimenter for the next trial. At the completion of each vision condition block of trials, participants answered the following attention focus question by ticking a box in a booklet: “Where did you focus most on the last block of set shot kicks, on the target line or on my technique?.” Self-report of attentional focus has been previously used in the literature (e.g., Wang et al., 2021). Participants were given 1–2 min break between vision condition blocks of trials to complete this question and for a small rest period.

### 2.3 Data analysis

The four primary dependent measures were mean absolute, constant, and variable error from the target as well as kick score. Absolute error from the target on the video record was determined by calculating the distance in centimeters from the middle of the ball to the closest point on the vertical target in the middle of the goal when the ball hit, or was alongside, the target (Magill and Anderson, 2021). Constant error was calculated as a signed deviation of absolute error with negative and positive values indicating kick error to the left and right of the ribbon, respectively (Magill and Anderson, 2021). Variable error was calculated as the standard deviation of constant error (Magill and Anderson, 2021). Kick score was determined by whether the ball traveled through the simulated goal or point scoring zones. The secondary dependent measure was external (i.e., on the target line) or internal (i.e., on technique) focus of attention. Five separate Generalized Estimating Equation (GEE) analyses were run to compare the dependent variables with vision condition as a fixed effect and individual participants included in the model as a repeated factor. As target error is a continuous measure, a linear GEE model was used and residuals were checked to be within ±3.29 for skewness and kurtosis (Ballinger, 2004; Field, 2013). For kicking accuracy and attention focus, which are dichotomous variables, binary logistic GEE models were used. These models do not require normality of residuals (Ballinger, 2004). GEEs were used as they allow for the correct modeling of repeated observations and can accommodate non-normally distributed data (Ghisletta and Spini, 2004). Alpha level was set at 0.05 and Holm-Bonferroni corrections were applied for follow-up post-hoc comparisons (Ballinger, 2004; Ghisletta and Spini, 2004).

## 3 Results

### 3.1 Target error

**Figure 2:**
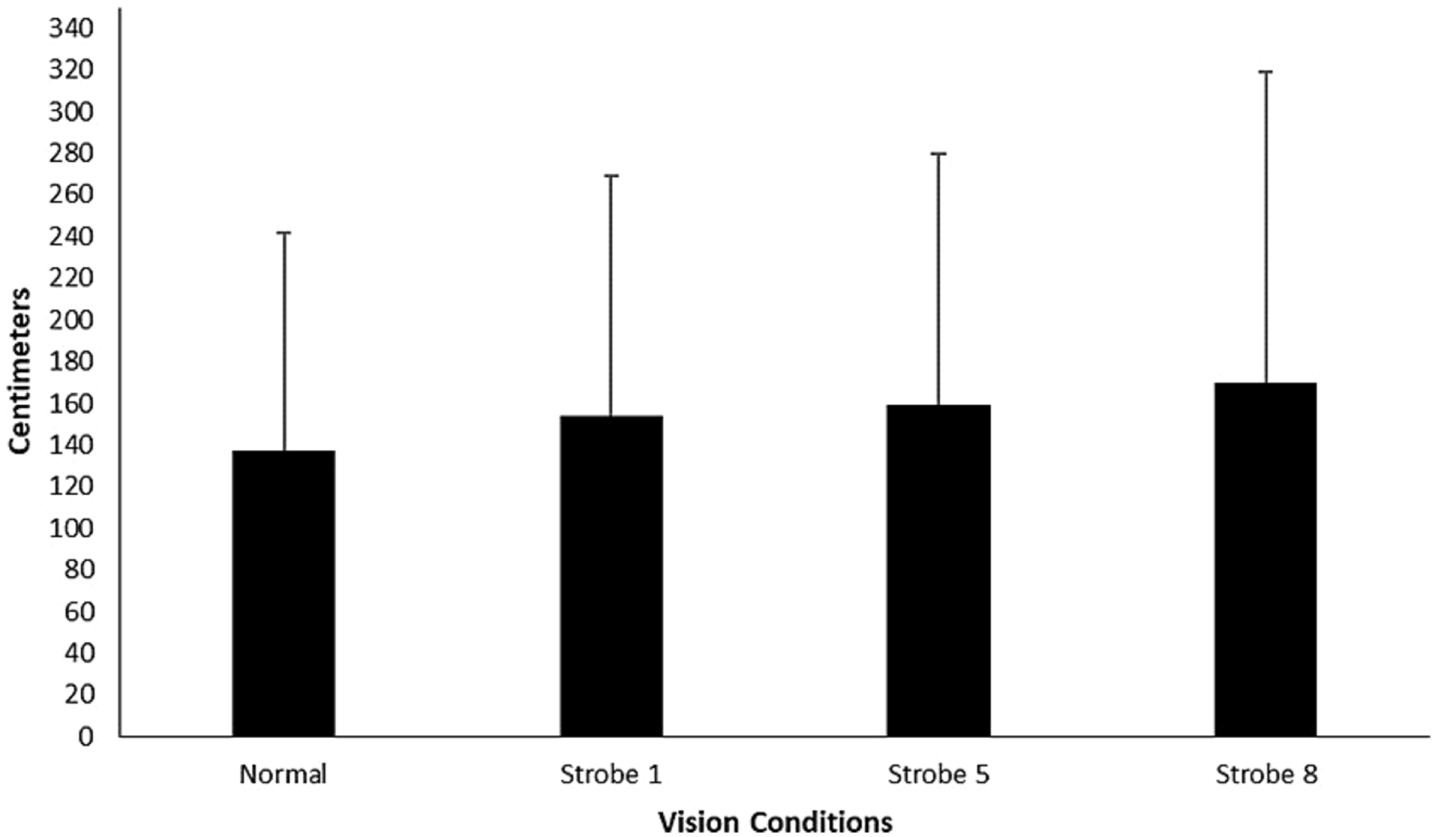
Absolute set shot kick error from the target (zero) in the goal under normal and stroboscopic vision conditions. Error bars represent standard deviations.

Figure 2 graphs the kick mean absolute error from the vertical target in the goal relative to normal and stroboscopic vision conditions. GEE found no significant difference in set shot kick mean absolute error across normal and stroboscopic vision conditions, *ꭓ*^2^ (3) = 3.45, *p* = 0.328. There was also no significant difference across stroboscopic vision conditions for mean constant error (Normal vision, *M* = 32.31 cm, SD = 170.16 cm; Strobe 1, *M* = −0.73 cm, SD = 193.01 cm; Strobe 5, *M* = 46.78 cm, SD = 194.28 cm; Strobe 8, *M* = 32.19 cm, SD = 223.87 cm), *ꭓ*^2^ (3) = 2.68, *p* = 0.445, and variable error (Normal vision, *M* = 144.14 cm, SD = 40.86 cm; Strobe 1, *M* = 160.62 cm, SD = 58.74 cm; Strobe 5, *M* = 170.47 cm, SD = 55.75 cm; Strobe 8, *M* = 183.95 cm, SD = 81.39 cm), *ꭓ*^2^ (3) = 4.76, *p* = 0.190.

### 3.2 Kicking accuracy

**Figure 3:**
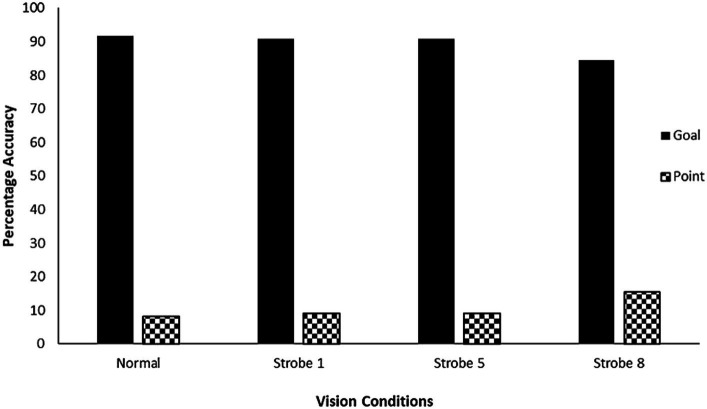
Absolute percentage of goals and points scored from the set shot kicks under the normal and stroboscopic vision conditions. Error bars are not plotted, as these are absolute values.

Figure 3 graphs the absolute percentage of goals and points scored relative to normal and stroboscopic vision conditions. GEE for kicking accuracy was significant, *ꭓ*^2^ (3) = 8.08, *p* = 0.044. However, post-hoc comparisons revealed no significant difference in the proportions of goals and points scored across normal to stroboscopic vision conditions (*p* > 0.05).

### 3.3 Attentional focus

**Figure 4:**
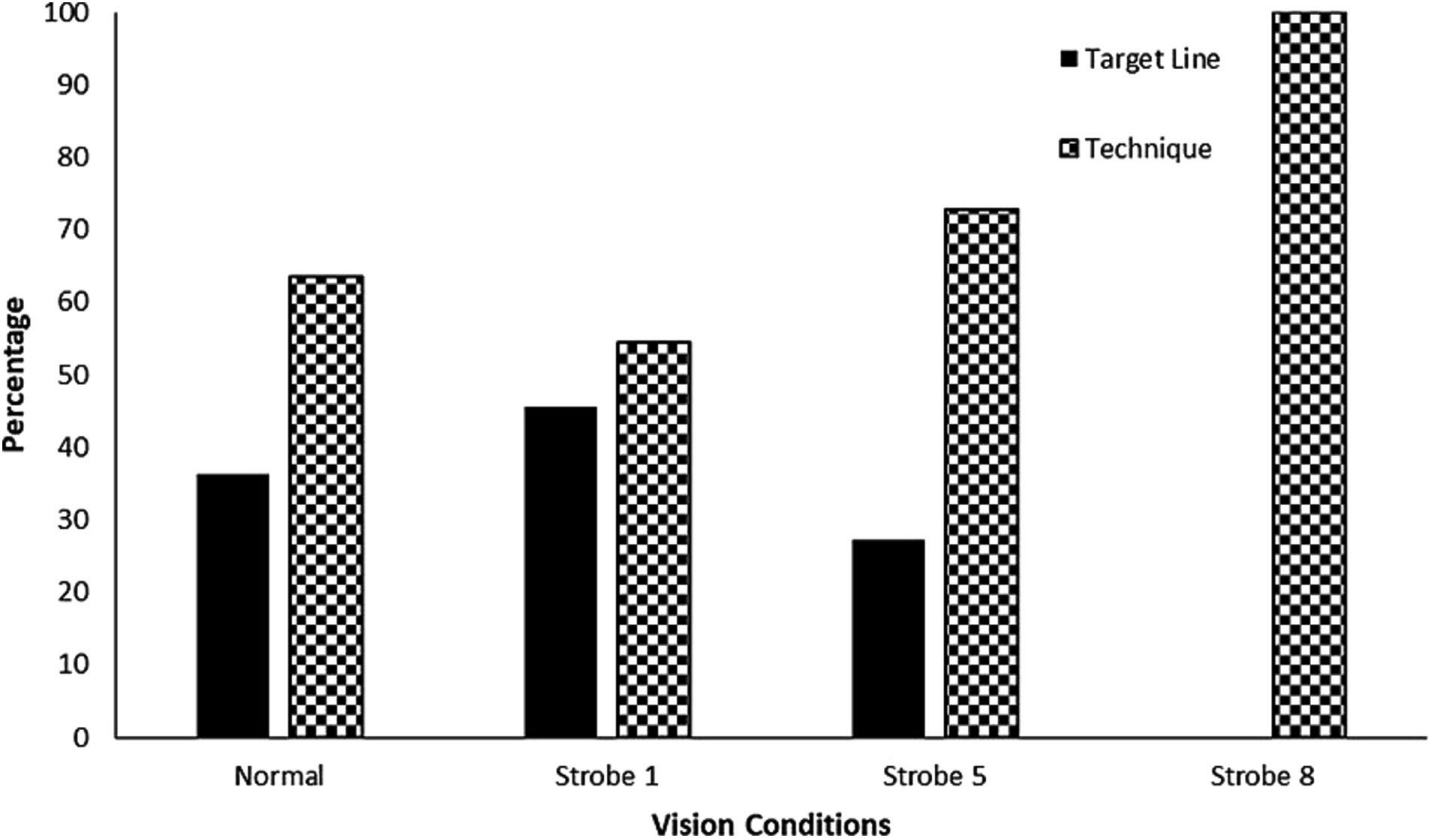
Absolute percentage of external and internal focus of attention under normal and stroboscopic vision conditions. Error bars are not plotted, as these are absolute values.

Figure 4 graphs the absolute percentage of reported external and internal focus relative to normal and stroboscopic vision conditions. GEE for attention focus was significant, *χ*^2^ (2) = 2930.72, *p* < 0.001. The source of the main effect was that Strobe 1 was significantly different to Strobe 8, *p* = 0.015. There was no significant difference in the proportions of attention focus between normal vision and Strobe 1, 5, and 8, as well as between Strobe 1 and 5, and Strobe 5 and 8, *p* > 0.05. Although, differences in proportions of attention focus approached significance between normal vision and Strobe 8 conditions, *p* = 0.061.

## 4 Discussion

This study set out to investigate how skilled athletes use visual-perceptual information to guide action in a complex exemplar skill of the set shot kick in Australian football. Stroboscopic glasses worn by the performer allowed manipulation of vision from normal to intermittent occlusion cycling conditions that interrupted the continuous perception-action cycle. The findings contribute to the growing evidence of whether a continuous uninterrupted perception-action cycle is necessary for skilled performers to utilize visual information to guide action. In addition, the study provided evidence of athletes reporting shifts in their attentional focus when moving from normal vision to intermittent occlusion conditions, and how these shifts influenced their skill performance. Collectively, the findings contribute to furthering theoretical knowledge of visual regulation of action and associated attention focus of skilled perceptual-cognitive-motor skill.

Based upon open and closed-loop theory accounts of perceptual-motor control, the first hypothesis predicted that temporal samples of visual information would be sufficient to guide set shot kick performance. Alternatively, based upon perception-action cycling accounts of motor control, the second hypothesis predicted that interruption of the vision-to-action cycle would decrease set shot kick performance. The first line of evidence revealed that kick target absolute, constant, and variable error (or local accuracy) did not change significantly across normal to stroboscopic vision conditions (see Figure 2). However, descriptively, variable error increased across normal vision to strobe 8 conditions suggesting that consistency of kick accuracy decreased as vision occlusion increased. A possible explanation is that increased vision occlusion may have resulted in minor delays of mapping temporal samples of visual information to limb positioning during the kicking action (Elliott and Bennett, 2020). Nonetheless, support for hypothesis one, but not two, was forthcoming. Skilled athletes in our study could use temporal samples of visual information to guide their run toward the mannequin to a kick position and guide the ball with their hand to be intercepted with their foot, to direct the ball over the mannequin at a target approximately 25 meters away from where it was kicked. This is consistent with the previously reported use of temporal samples of visual information to time positioning of the limb in one handed catching (Elliott and Bennett, 2020). Our study goes beyond that literature to demonstrate that temporal samples of visual information for action control is in line with previous expertise studies that have reported temporal pick-up of visual information for whole body positioning to intercept a ball in striking sports (Abernethy et al., 2001; Müller et al., 2009). Therefore, a continuous intact perception-action cycle does not seem necessary for local aiming (target error) at a target.

The second line of evidence supporting hypothesis one, and not hypothesis two, is that the proportion of goals and points scored did not change significantly across normal to interrupted vision conditions (see Figure 3). This indicates that temporal sampling of visual information to guide action did not impede the spatiotemporal interception of the ball by foot during the kick action for its resultant goal scoring opportunity. Again, this evidence is in line with temporal sampling of visual information to guide global performance in terms of the number of balls caught in the one handed catching literature (Elliott and Bennett, 2020). This evidence is also consistent with temporal sampling of object flight to intercept a ball accurately with a bat, or a puck with a glove or stick, respectively (Müller et al., 2009; Panchuk and Vickers, 2009). It is possible that temporal visual sampling may have been used to make fine adjustments to the kick run-up and/or ball-foot interception, as reported in striking sports (Müller et al., 2009; Panchuk and Vickers, 2009), but this requires investigation in follow-up studies. Our findings are not consistent with previous studies that reported decrements to performance in junior and amateur players (Fransen et al., 2017; Beavan et al., 2020). A possible reason is that at junior and lesser skilled levels, players may not have developed the capability to predict future events based upon temporal visual samples and require continuous visual feedback (Fransen et al., 2017). This also requires further investigation in future work where skill level is manipulated in the same task. Therefore, again, a continuous perception-action cycle was not necessary to maintain the global accuracy requirements of the motor skill in this study for skilled participants.

Broadly, internal focus of attention on a movement pattern has been associated with decreased performance compared to external focus upon environmental information or action effects (Wulf et al., 2001). Hypothesis three predicted that a change in self-reported attention focus from external to internal would result in a decrement to performance. As mentioned, there was no accompanying significant increase in local accuracy (target errors) or significant decrease in global accuracy (goals scored) across vision conditions (see Figures 2, 3), despite there being a significant change to predominantly internal focus of attention as vision occlusion increased from Strobe 1 to 8 (see Figure 4). There are two possible explanations for this. First, perhaps skilled athletes in our study had the intricate capability to switch attentional focus at a critical moment of skill phase (e.g., ball-foot impact) to maintain performance as has been reported in golf putting (Wang et al., 2021). Our questions probed allocation of attentional focus in a dichotomous perspective, which did not account for attentional switching. Second, participants’ reported focus on technique may have been on proprioceptive control (Elliott and Bennett, 2020), rather than conscious focus or conscious control upon isolated components of the movement pattern in the kick. Given the intermittent nature of occlusion-to-vision cycling of the stroboscopic glasses, it is possible that temporal samples of visual information created a shared attention focus between external environmental and internal technique information despite written reports (Herrebrøden, 2022). Accordingly, intermittent sampling of visual information for action may be accompanied by dynamic attentional focus shifts, rather than previous conceptualization as static (or fixed) attentional focus during skill execution (Abdollahipour et al., 2016; Saemi et al., 2017). Collectively, these explanations warrant further research investigation, particularly since it is possible to shift attentional focus through manipulation of vision using stroboscopic glasses.

The theoretical implication of this study is that it contributes to furthering knowledge of visual regulation of complex interceptive action. The findings contribute to a growing body of evidence in open sports skills (Abernethy et al., 2001; Panchuk and Vickers, 2009; van Soest et al., 2010) that temporal samples of visual information also appear sufficient to maintain skilled performance in a closed sports skill. A reason why temporal samples of visual information are sufficient for action control is because a performer is faced with a multitude of sensorimotor information. That is, at any given time of complex open or closed sports skills, performers are faced with an array of visual, proprioceptive, and haptic information (Elliott and Bennett, 2020). The neuromotor system can only use a select amount of this sensorimotor information at particular instances in time to predict future environmental states and couple body action to complete the skill goal (Elliott and Bennett, 2020). Selection of sensorimotor information may also require dynamic shifts in attentional focus to maintain performance. Therefore, knowing that temporal samples are sufficient for action control presents opportunities for challenging practice and selective guidance of sensory information to accelerate perceptual-cognitive-motor skill.

Standard practice of interceptive sports skills is typically conducted under normal uninterrupted vision conditions. While this is representative of the sensory condition that motor skills are performed under in competition, there has been recent interest of whether visual occlusion paradigms can challenge visuomotor skill to accelerate performance. Initial intervention studies using stroboscopic vision training have reported improvements to skate and pass accuracy in ice-hockey (Mitroff et al., 2013), and that it might contribute to batting practice performance in baseball (Liu et al., 2020). The findings from our study suggest that stroboscopic glasses could be used as a screening tool for sports skills. For example, normal and stroboscopic vision conditions could be designed to determine whether athletes can use temporal visual samples to execute skills and how attentional focus is allocated during skill execution. This would provide information to guide design of visual-perceptual and/or proprioceptive-based attention focused training interventions to enhance skill performance (Mulligan and Hodges, 2014; Brenton et al., 2019).

## 5 Conclusion

The task designed for this study interrupted the perception-action cycle during a complex interceptive skill and found that temporal sampling of visual information, as well as combined internal and external focus of attention were sufficient to maintain performance. This indicates that a continuous intact perception-action cycling is not necessary for proficient perceptual-cognitive-motor skill. A potential limitation is that the number of kicks per vision condition was 10. However, our approach was to reduce any potential for athlete injury from increased number of kicks in a single session by ensuring that the statistical analysis was adequately powered. Future research could build upon our study in two main ways. First, written report of attentional focus under normal and stroboscopic vision could be expanded to probe whether external focus (i.e., proximal or distal, initial ball kick path or goal location, respectively), internal focus (technique or proprioceptive ‘feel’ of the movement) or combinations of both contribute to maintaining interceptive skill performance. This would contribute to concerns raised in the literature that all forms of internal focus may not be a limiting factor to performance (Herrebrøden, 2022). Second, an intervention study including normal and stroboscopic vision conditions is needed to determine whether complex open and closed motor skill performance can be accelerated. While initial attempts have been made to probe this question, stroboscopic training has been mixed with general visual skills training making it difficult to delineate causality (Liu et al., 2020). Both these future research directions will contribute to advancing the theoretical underpinning of visual control of action and how it can be enhanced.

## Data availability statement

The datasets presented in this article are not readily available because athlete data cannot be shared. Requests to access the datasets should be directed to sean.muller@federation.edu.au.

## Ethics statement

The studies involving humans were approved by Federation University Human Research Ethics Committee (approval number E21-004). The studies were conducted in accordance with the local legislation and institutional requirements. The participants provided their written informed consent to participate in this study.

## Author contributions

SM: Conceptualization, Data curation, Formal analysis, Writing – original draft. BB: Conceptualization, Project administration, Writing – original draft, Data curation. KM-B: Conceptualization, Data curation, Formal analysis, Writing – original draft. CM: Conceptualization, Writing – original draft, Methodology.
